# Hand-Book of Materia Medica, Pharmacy and Therapeutics

**Published:** 1889-03

**Authors:** 


HAND-BOOK OF MATERIA MEDICA, PHARMACY AND THERAPEUTICS, compiled for the use of students preparing for examination. By Cuthbert Bowen, M. D., B. A., editor of "Notes on Practice." Published by F. A. Davis, Philadelphia, 1888. Price $1.40.

This is a handsome little book containing 350 pages. It aims at furnishing candidates for examination with a resume of those points in Materia Medica, Pharmacy and Therapeutics with which they must be familiar in order to meet the requirements of the faculty at any reputable school. The information is imparted by questions and answers which form gives the greatest amount of knowledge in the fewest words. Ordinarily such books are not to be encouraged, but the present volume, not intended to take the place of more extensive works, is a real help in the proper place. —B.

